# Nonantibiotic-driven evolution reveals rare but predictable routes to broad antibiotic resistance

**DOI:** 10.1128/mbio.01168-26

**Published:** 2026-08-03

**Authors:** Carmen Li, Amir Mitchell

**Affiliations:** 1 Department of Systems Biology, University of Massachusetts Chan Medical School 12262, Worcester, Massachusetts, USA; Universiteit Gent, Gent, Belgium

**Keywords:** drug resistance evolution, antibiotic resistance, multidrug resistance, *Escherichia coli*, efflux pumps, genome analysis

## Abstract

Many medications not prescribed to treat infectious diseases have antibacterial activity at physiologically relevant concentrations, raising the risk that chronic administration of such nonantibiotics may inadvertently select for resistance in the host microbiome. However, how frequently such exposures select for adaptations that impact broad drug resistance, including to antibiotics, remains unclear. Here, we systematically evolved *Escherichia coli* under exposure to 40 antibiotics and nonantibiotics and profiled the cross-resistance of the drug-adapted strains to 21 antibiotics representing all major classes. Our measurements revealed that most drug-adapted strains did not become multidrug resistant. However, five nonantibiotics and three antibiotics emerged as exceptions and were repeatedly selected for broad antibiotic resistance. Whole-genome sequencing of all 168 evolved strains revealed that changes in the regulation of efflux pumps repeatedly underlay broad drug resistance and converged into two key regulatory genes, *acrR* and *lon*. Our work suggests that although inadvertent antibiotic cross-resistance is rare, specific nonantibiotics can still potentially pose a risk for the emergence of multidrug resistance.

**IMPORTANCE:**

Many medications not typically prescribed to treat infectious diseases have potent antimicrobial activity at physiological concentrations. This anti-bacterial activity raises concern that long-term administration of such nonantibiotics might unintentionally select for multidrug resistance, including resistance to antibiotics. Using *Escherichia coli*, we show that in most cases, these nonantibiotics do not broadly select for resistance to antibiotics *in vitro*. However, we identified five nonantibiotics that repeatedly selected for resistance to multiple antibiotics through a shared mechanism of action—upregulation of the multidrug efflux pump AcrAB-TolC. These findings highlight that while the overall risk is low, certain nonantibiotics may still contribute to the emergence of multidrug resistance. Identifying these high-risk drugs can help guide safer prescribing practices and inform strategies to limit the spread of antibiotic resistance.

## INTRODUCTION

Many drugs not prescribed to treat bacterial infections, called nonantibiotics hereafter, can inhibit bacterial growth. Multiple studies have shown that these compounds are associated with changes in microbiome composition (1–3). A systematic *in vitro* screen revealed that approximately one-quarter of the tested nonantibiotics exhibit antibacterial activity at physiologically relevant concentrations (4). These findings indicate that nonantibiotics impose measurable selective pressures on bacteria and may drive some of the compositional changes observed in the host microbiome in association with nonantibiotics. While previous studies of dozens of nonantibiotics suggested that they operate differently from standard antibiotics (5–7), additional works revealed that nonantibiotics can still sometimes select for resistance mechanisms that provide broad antibiotic resistance (6, 8–11). A key question that remains unanswered is how frequently nonantibiotics select for multidrug resistance and whether this frequency is considerably elevated in comparison to standard antibiotics. These questions are particularly relevant, given the widespread and often chronic use of nonantibiotics, which may create sustained selection pressures on the host microbiome.

The evolution of bacterial resistance under drug exposure has been extensively studied in the context of antibiotics. Laboratory evolution experiments have shown that resistance can emerge rapidly and extend beyond the selecting drug. Large-scale studies mapping collateral sensitivity and cross-resistance networks have demonstrated that adaptation to one antibiotic can alter susceptibility to others (12–14). These studies highlighted that multiple paths can lead toward resistance, with some adaptations impacting resistance beyond the specific drug used during the evolution experiments (15). Additional works have shown that cross-resistance frequently arises through mutations in regulatory networks that control multidrug efflux systems (16, 17). Collectively, these studies provide an established framework for understanding how antibiotic exposure drives resistance and cross-resistance. In contrast, despite growing evidence that nonantibiotics can impose selective pressures on bacteria, the evolutionary consequences of prolonged exposure to these drugs, including their potential to drive antibiotic cross-resistance, remain underexplored.

Emerging evidence suggests that bacteria can adapt to nonantibiotics, altering their susceptibility to these compounds. For example, we have shown that adaptation of *Escherichia coli* to the anti-cancer chemotherapies gemcitabine (7) and 5-fluorouracil (5) occurs rapidly and can alter bacterial fitness and drug responses in host-associated contexts. These findings demonstrate that nonantibiotics can act as selective agents that drive bacterial evolution. In some cases, this adaptation can extend beyond the selected drug and lead to multidrug resistance. For instance, exposure to the antipsychotic drug quetiapine has been shown to select for mutations in a repressor of a multidrug efflux pump, thereby conferring resistance to multiple antibiotics (8). Similarly, multiple studies have shown that antidepressants select for broad antibiotic cross-resistance (6, 9–11). Recent studies on painkillers (18) and an antidepressant (10) highlighted another potential connection between nonantibiotics and antibiotic resistance by demonstrating that they can increase the mutation frequency in bacteria exposed to these drugs. Together, these works demonstrate that nonantibiotics can promote cross-resistance phenotypes, but the extent and generality of this effect remain unclear.

Here, we systematically evaluated the potential for nonantibiotics to drive antibiotic cross-resistance by directly comparing them to antibiotics under the same experimental conditions. We evolved *E. coli* under prolonged exposure to a panel of 21 nonantibiotics drugs and 19 antibiotics. We then determined the resistance profiles of the evolved bacteria, relative to the ancestral wild-type, across multiple antibiotic classes. This approach allowed us to directly quantify the frequency and the extent to which adaptation to individual drugs leads to broad antibiotic resistance. We found that in most cases, adaptation to nonantibiotics did not result in broad antibiotic cross-resistance. However, a subset of both nonantibiotics and antibiotics consistently selected for multidrug resistance, relative to the ancestor, through mutations in two key regulatory genes, *acrR* and *lon*, which regulate the expression of the multidrug efflux pump AcrAB-TolC. These findings demonstrate that while the overall risk of cross-resistance is limited, specific nonantibiotics can reproducibly drive the emergence of multidrug resistance.

## RESULTS

### Resistance emerges under the selection of antibiotics and nonantibiotics

**Fig 1:**
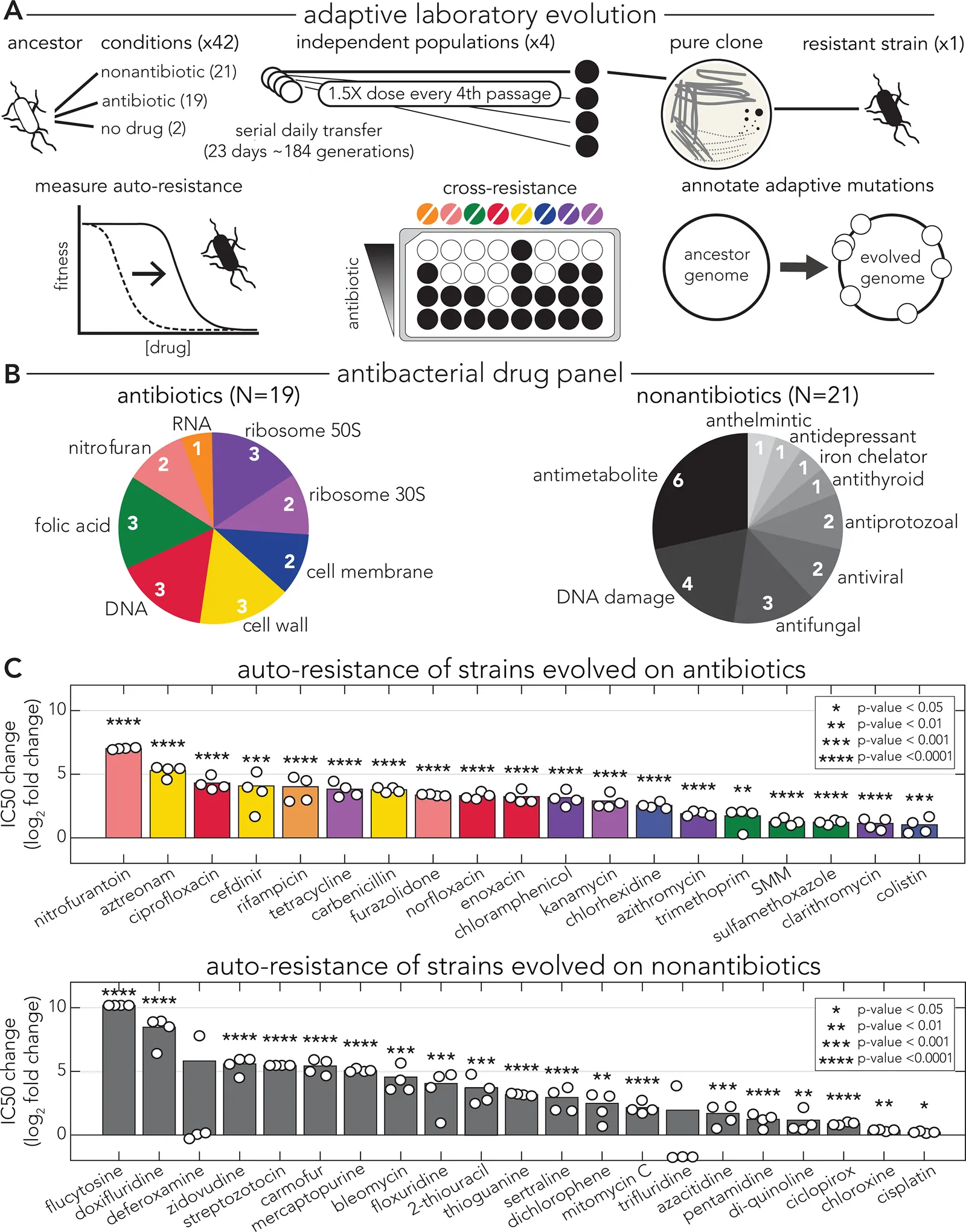
Auto-resistance rapidly evolves under the selection of a panel of 40 drugs. (**A**) Outline of experimental approach. We evolved four independent replicates of *E. coli* on each drug using the serial transfer evolution protocol over 23 days. We isolated a single pure clone from each independently evolved population and evaluated its resistance against the drug it evolved on (auto-resistance) and its resistance against a panel of 21 representative antibiotics (cross-resistance). We sequenced the genome of the evolved strain and annotated adaptive changes by reoccurrence across same-drug replicates. (**B**) Drug classes of the 40-drug panel we used for evolution experiments. (**C**) The change in auto-resistance in evolved strains. The bars show the relative concentration required to inhibit growth by 50% (IC50) averaged across evolved replicates relative to the ancestral strain. Auto-resistance of individual strains is shown as circles. The star indicates statistical significance in a one-tailed Welch’s *t*-test. Sulfamonomethoxine is abbreviated as SMM, and diiodohydroxyquinoline is abbreviated as di-quinoline.

To determine the risk in evolved cross-resistance, we followed the widely used serial transfer approach to select for evolved drug resistance in microorganisms (19). While these experiments are *in vitro*, they can still provide insight into the molecular mechanisms underlying adaptive resistance within short evolutionary timescales. We used this protocol to evolve multiple replicates of an *E. coli* laboratory strain starting at a sub-inhibitory concentration for the 40 drugs. For each drug, we evolved four independent populations over a period of 23 days. We isolated a single pure clone from each evolved population for downstream analysis. We then determined the clone’s resistance to the drug used for evolution (auto-resistance) and cross-resistance to a panel of antibiotics representative of all major classes. Our measurements of resistance and sensitivity are relative to the ancestor and are independent of clinical breakpoints used to define antibiotic susceptibility. Finally, we performed whole-genome sequencing to identify the adaptive mutations. Figure 1A shows an overview of the research approach of our study.

The drug panel we used for the evolution experiments included 19 antibiotics, representing the 8 major antibiotic classes and 21 nonantibiotics (Fig. 1B). Nonantibiotics were selected based on our previous observation that they exhibit antibacterial activity and have diverse host-targeted mechanisms (6). To quantify auto-resistance, we measured the growth of the evolved strains relative to the ancestor strain across a wide range of drug concentrations and inferred the concentration that inhibits growth by 50% (IC50); 154 out of 160 strains evolved auto-resistance within the experimental timeframe (Fig. 1C and Table S1, sheet 1: Auto-resistance). We observed differences in the resistance levels between drugs, but the resistance levels were largely reproducible across independently evolved replicates (Fig. 1C). For example, the strains evolved under nitrofurantoin exhibited the highest resistance among antibiotic-selected strains, whereas evolution under the antibiotic of the same class, furazolidone, resulted in an approximately 2-fold lower resistance. Average auto-resistance levels that were statistically significant varied dramatically between drugs and ranged between 1.2-fold and over 1,000-fold. For two drugs, deferoxamine and trifluridine, only one replicate evolved resistance. We also observed that evolution on some drug classes resulted in different levels of auto-resistance (Fig. S1). In summary, resistance evolved rapidly and reproducibly across diverse drugs under the sustained selection of our evolution protocol.

### Characterization of cross-resistance

Having established that auto-resistance evolved under selection, we next measured antibiotic susceptibility. Since the number of measurements required for evaluating cross-resistance is over an order of magnitude larger than those needed for auto-resistance, we relied on a method that can work at the scale needed. We measured susceptibility by following the European Committee on Antimicrobial Susceptibility Testing (EUCAST) guidelines (20), with a method we previously developed for high-throughput broth microdilution (BMD) assays (21). Specifically, we grew the evolved and ancestor strains in 96-well plates for 24 h in serial antibiotic dilutions. We tested susceptibility against a panel of 21 antibiotics representative of the eight major antibiotic classes. We applied automated image analysis using a pre-trained convolutional neural network to quantify the highest drug concentration at which robust growth is observed (maximum growth concentration [MGC]). We used this metric since it better captures small changes in drug resistance (in contrast to standard MIC measurements). We compared the auto-resistance measurements obtained from dose-response assays with those obtained from the high-throughput BMD methods and found that they were consistent with each other (Pearson *r* = 0.67, *P* = 5.25 × 10^−11^; Fig. S2). Overall, this indicates that the BMD assay robustly captures differences in resistance across conditions.

**Fig 2:**
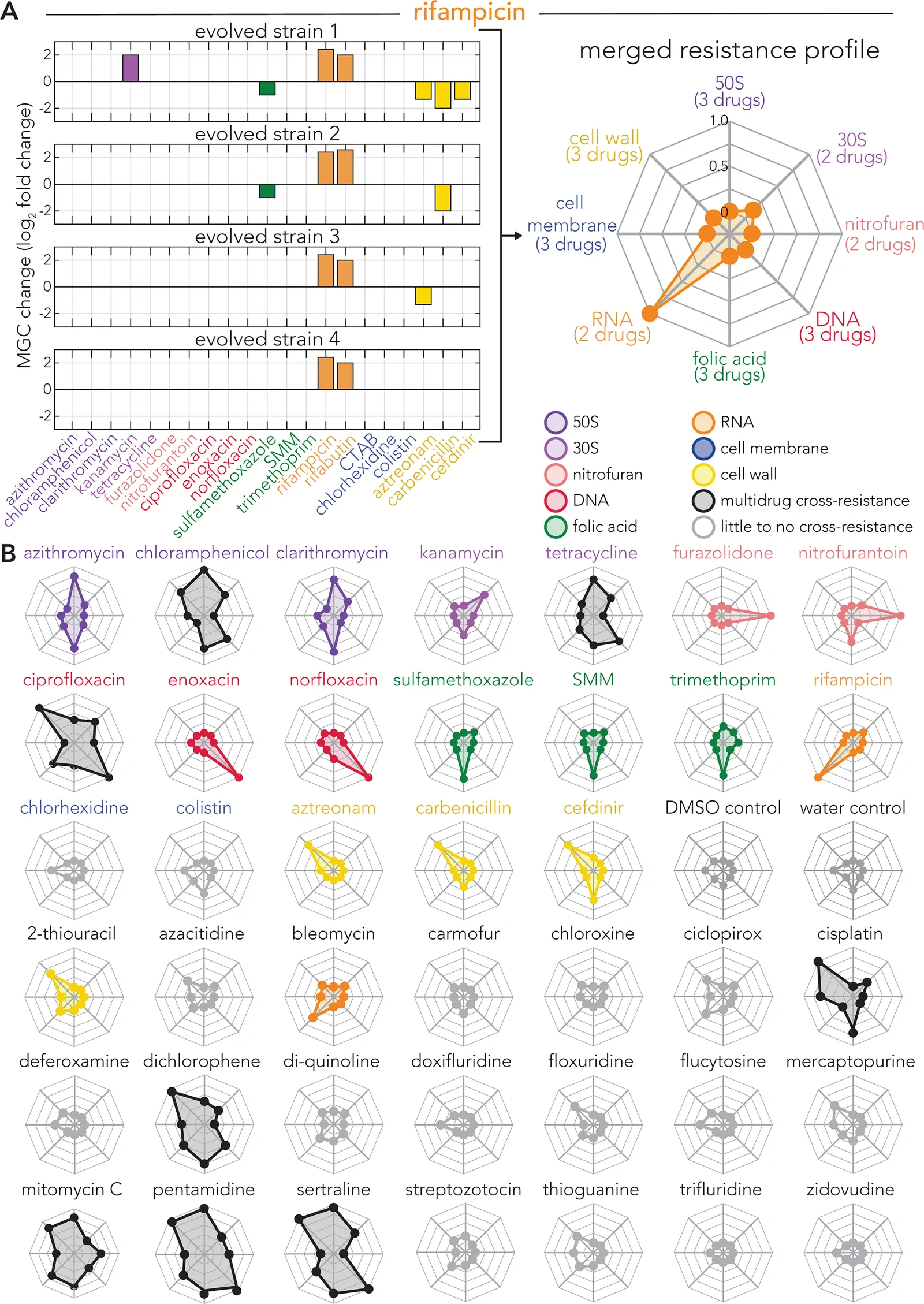
Cross-resistance measurements in evolved strains reveal most adaptation trajectories resulted in single-class resistance profiles.** (A**) A representative example of an evolved resistance profile (rifampicin-evolved strains). The bars mark the change in MGC against the antibiotic panel for each rifampicin-evolved strain, relative to the ancestor strain. The bars mark the mean of three replicates (left panel). Cetrimonium bromide is abbreviated as CTAB. The right panel shows a summary radar plot of the resistance profile inferred from the cross-resistance measurements (Materials and Methods). (**B)** The resistance profile of strains evolved on the forty drugs. The color of the web denotes the antibiotic class the strains were most resistant to, and the color of the drug name marks the class of the drug used for evolution. The gray-filled web indicates resistance to three or more antibiotic classes (multi-antibiotic resistance). The empty web indicates no antibiotic resistance. Sulfamonomethoxine is abbreviated as SMM, and diiodohydroxyquinoline is abbreviated as di-quinoline.

To enable a direct comparison between the cross-resistance patterns, we consolidated resistance measurements into compact radar plots. Figure 2A shows an example of strains evolved on rifampicin. In agreement with previous auto-resistance measurements (Fig. 1C), all the strains evolved on rifampicin become rifampicin-resistant. However, cross-resistance measurements further revealed the strains were also resistant to rifabutin, an antibiotic from the same class, and some strains became either sensitive or resistant to antibiotics from other classes. To get a compact representation of the resistance profile for strains evolved on rifampicin, we combined the resistance of all four evolved strains to a single radar plot showing the relative level of resistance normalized for each antibiotic class (Fig. 2A, right panel).

In total, we obtained 10,836 susceptibility measurements across all antibiotics, concentration ranges, strains, and replicates (Table S1, sheet 2: Cross-resistance). Figure 2B summarizes the cross-resistance results using the compact radar plots. As expected, the strains evolved on antibiotics became predominantly resistant to antibiotics of the same class (the color of the web matches that of the drug name in Fig. 2B). However, some antibiotics (azithromycin, clarithromycin, kanamycin, nitrofurantoin, and cefdinir) selected for cross-class resistance. A notable exception to this pattern was observed for the class of cell membrane disruptors, chlorhexidine and colistin. For these drugs, the evolved strains did not become resistant to antibiotics in the same class. This difference likely reflects variation in their mechanisms of membrane disruption, resulting in distinct resistance mechanisms. Chlorhexidine disrupts by binding broadly to the cell wall (22), while colistin disrupts by binding only to the lipid A component of the outer membrane (23). Unsurprisingly, for nonantibiotics, many of the strains did not develop resistance or sensitivity to antibiotics (bottom panel of Fig. 2B). We defined multidrug resistance as resistance to three or more antibiotic classes. We observed that selection by three antibiotics and five nonantibiotics resulted in multidrug resistance (marked by gray-filled webs in Fig. 2B). For antibiotics, the selection of multidrug resistance was not specific to an antibiotic class. Overall, multidrug resistance was relatively rare for both drug groups, with 15.8% for the tested antibiotics and 23.8% for the tested nonantibiotics. We further tested if the level of auto-resistance was associated with the emergence of multidrug resistance (Fig. S1). A statistical test rejected this association.

In addition to resistance patterns, we examined the sensitivity profiles (Fig. S3). Most antibiotic- and nonantibiotic-evolved strains did not acquire sensitivity to other antibiotics relative to the ancestor strain. The predominant sensitivity was to the cell wall synthesis inhibitor class. However, the water control condition showed a similar sensitivity, suggesting that this effect possibly arises from adaptation to the serial transfer protocol of the evolution experiment.

Taken together, our systematic measurement of antibiotic resistance across all evolved strains revealed similar adaptation trends for both antibiotics and nonantibiotics. In the majority of cases, evolved resistance was restricted to the specific drug (or drug class) used for selection. This restricted resistance profile suggests that evolved resistance was mostly attributed to adaptation related to the drug-specific mechanisms. However, despite this overall trend, clear instances of cross-resistance were observed for both antibiotics and nonantibiotics. The frequency of such events was similar in the two drug groups and was consistent across independently evolving biological replicates.

### Evolved mechanisms of drug resistance

Drug sensitivity measurements revealed that most of the evolved strains adapted and became drug resistant. To determine the genetic mechanisms underlying resistance, we sequenced the genomes of the evolved strains by short-read sequencing (Illumina, 150 bp paired-end reads). We then used the breseq software (24) to identify mutations in each genome by comparing it to the reference genome of our lab strain (NC_00913). Mutations common to all sequenced strains were ignored, as they reflect differences between the ancestor strain in our experiment and the reference strain. In total, we sequenced 160 genomes of drug-evolved strains and eight genomes from strains serially transferred without any drug as controls. The median coverage of genome sequencing was 81-fold.

**Fig 3:**
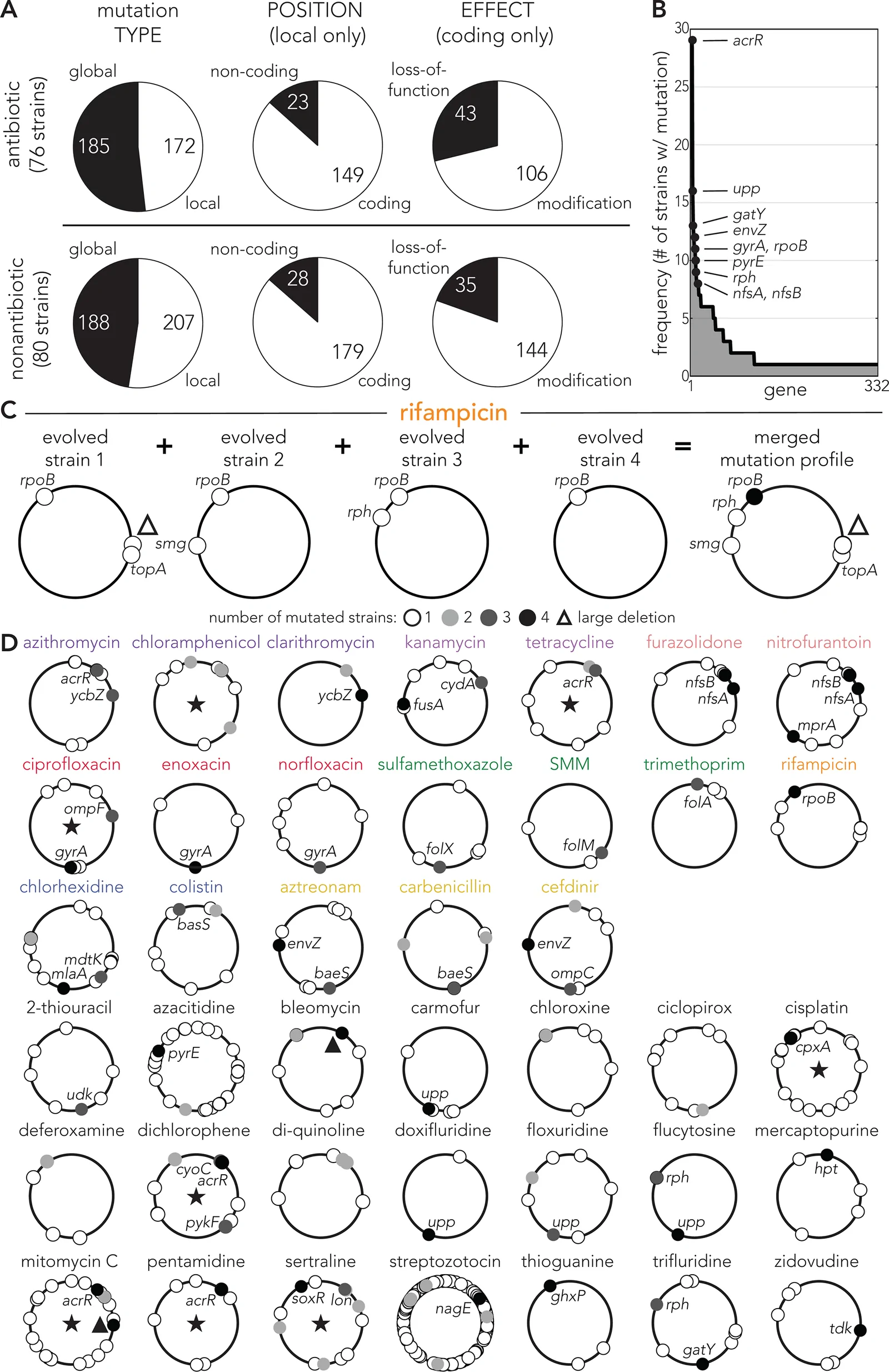
Whole genome sequencing of evolved strains uncovers repeatedly mutated genes within drug replicates and across drugs. (**A)** Characterization of the mutation profiles in strains evolved on antibiotic and nonantibiotic drugs. The pie charts mark the type, the position of local mutations, and the effect of local mutations on coding regions cataloged across all evolved strains (streptozotocin-evolved strains are not included). Mutations that clearly affect only a small region are annotated as local mutations (SNPs, small insertions, mobile element insertions, and small deletions). Mutations that clearly disrupt the reading frame are annotated as loss-of-function (nonsense SNPs, frameshift mutations, and small deletions). (**B)** The frequency of mutations in specific genes counted across all drug-evolved strains (streptozotocin-evolved strains are not included). The top 10 mutated genes are labeled. (**C)** A representative example of mutations in same-drug replicates (rifampicin-evolved strains). The four left circa plots show the position of mutations and the genes they affected in each evolved replicate strain. The right circa plot shows the combined representation of the mutation pattern across replicates. The color of the circles denotes the number of genomes that have a mutation in that gene. (**D)** The combined mutation patterns inferred from strains evolved on all 40 drugs. Genes that are mutated in three or more strains are labeled. The star in the middle of a circa plot indicates the strains were inferred to be multi-antibiotic resistant.

We first tested if the overall mutation patterns were similar in strains evolved on antibiotic and nonantibiotic drugs. Figure 3A summarizes the types of mutations we observed for 156 of the 160 drug-evolved strains. For nonantibiotic evolved strains, we excluded strains evolved on streptozotocin because it inhibits DNA synthesis and resulted in 216 single-nucleotide polymorphisms (SNPs) across the four evolved strains (highly skewing the statistics). We observed 357 mutations affecting 207 genes in antibiotic-evolved strains and 395 mutations affecting 168 genes in nonantibiotic-evolved strains. Both antibiotic- and nonantibiotic-evolved strains had similar percentages of mutations with local effects (single-nucleotide polymorphisms, small indels, and mobile element insertions) relative to global effects (large indels and genomic rearrangements). Of the mutations that have local effect, both antibiotic- and nonantibiotic-evolved strains had similar proportions of mutations located in the gene coding regions relative to non-coding regions. The proportion of mutations in the coding regions that could be clearly annotated as loss-of-function mutations was also overall similar between antibiotic- and nonantibiotic-evolved strains. Overall, we did not observe gross differences in the type, position, or effect of mutations between the two drug groups.

We reasoned that adaptive mutations can be annotated by identifying the genes that were repeatedly mutated across independently evolved strains (5–7, 25). First, we calculated the frequency of mutation, the number of strains with a mutation in that gene, for each of the 332 mutated genes in our sequenced genomes. Figure 3B shows the highly skewed frequency distribution of gene hits. We reasoned that since the largest antibiotic classes had 3 antibiotics and a total of 12 independently evolved strains, a gene hit frequency of 12 or above would highlight resistance mechanisms shared beyond a single drug class. Three genes passed this threshold. The most frequently mutated gene, found in 29 of the evolved strains, was *acrR*, a key regulator of the multidrug efflux pump AcrAB-TolC. The *upp* gene encoding an uracil phosphoribosyltransferase was mutated in 16 strains, likely reflecting its importance for resistance against different nucleotide analog chemotherapies (5). Finally, the *envZ* gene encoding a sensor histidine kinase was mutated in 12 strains, likely due to its role in modulating outer membrane pores and their impact on the permeability of multiple drugs (26).

To identify candidate drug-specific adaptive mutations, we focused on genes that were recurrently mutated in two or more independently evolved strains from the same drug treatment. Figure 3C shows the genomic location of the mutated genes for each individual strain evolved on the antibiotic rifampicin. Across these four replicates, only one gene, *rpoB,* was repeatedly mutated, with two different types of substitutions (Table S3). Substitutions in *rpoB*, the beta subunit of RNA polymerase, are known to confer resistance to rifampicin in *E. coli* (27), *Bacillus subtilis* (28), and *Mycobacterium tuberculosis* (29). Figure 3C summarizes the distribution of mutated genes for all strains evolved on each drug. The number of mutated genes varied widely between drugs, ranging from as few as two genes for clarithromycin, doxifluridine, and flucytosine to 205 genes for streptozotocin. The median number of mutated genes across all strains was seven. Despite this variability, across all drugs, there were only a handful of genes that were mutated across more than one replicate (typically 1–3). The full list of mutations and their annotations is provided in Tables S2 through S6.

Collectively, analysis of results from whole genome sequencing suggests that drug-specific evolved resistance was driven by a handful of adaptations (shared across independently evolved replicates of that drug) and that only a few general adaptation mechanisms were shared across multiple drug classes. Two of the three most common adaptations likely increase resistance by impacting intracellular drug availability, either by drug import (*envZ*) or drug removal (*acrR*).

### Multidrug resistance is associated with modified regulation of drug efflux pumps

**Fig 4:**
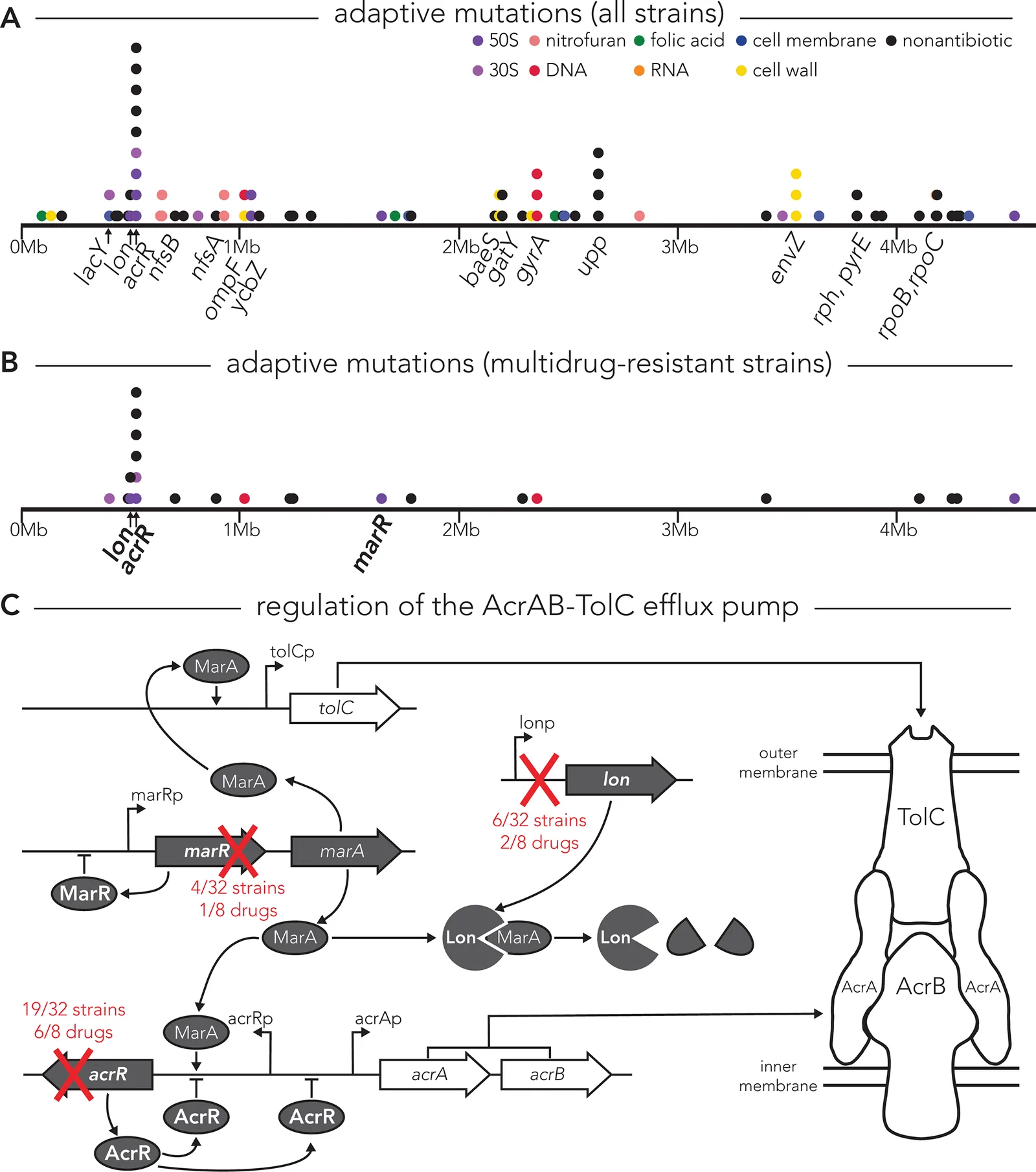
Evolution in multidrug-antibiotic resistant strains converged to changes in three regulators of the AcrAB-TolC efflux pump. (**A)** The genomic position of genes with adaptive mutations (observed in two or more replicate strains) for all drugs used for evolution. The color of the circle corresponds to the drug class, and the labeled genes mark adaptive mutations present in two or more different drugs. (**B)** The position of genes with adaptive mutations found only in multidrug-resistant strains. (**C)** A gene regulation diagram of the effector genes in the AcrAB-TolC efflux pump. Effector genes are marked in white and regulators in gray. The number of multidrug-resistant strains with mutations in each regulator and the number of drugs with adaptive mutations of the drugs that select for multidrug resistance in that regulator are marked in red.

Antibiotic cross-resistance patterns (Fig. 2B) suggested that most strains evolved on a single antibiotic became resistant to other antibiotics of the same class. We sought to determine whether resistance mechanisms uncovered by whole genome sequencing also converged within antibiotic classes. For this analysis, we identified genes with mutations common to multiple antibiotics from the same class. Figure 4A summarizes the set of genes with adaptive mutations determined by our analysis. We observed that for most classes of antibiotics, adaptive mutations converged to the same gene. Both nitrofuran-evolved strains (nitrofurantoin and furazolidone) harbor mutations in *nfsA*, a known first-step to resistance, and *nfsB*, a known second-step mutation for increased resistance (30). Many strains adapted to DNA gyrase inhibitors (ciprofloxacin, enoxacin, and norfloxacin) shared mutations in *gyrA*, which encodes a subunit of the DNA gyrase and is known to confer resistance to DNA gyrase inhibitors when mutated (31). Strains adapted to cell wall-targeting antibiotics (aztreonam and carbenicillin) repeatedly had mutations in *baeS*, the histidine kinase of the BaeSR two-component system involved in envelope stress adaptation (32). Strains resistant to cell wall-targeting antibiotics (aztreonam, carbenicillin, and cefdinir) also shared mutations in *envZ,* which is part of the EnvZ/OmpR two-component system modulating outer membrane diffusion pores. However, evolved strains from additional drugs (pentamidine and norfloxacin) also harbored *envZ* mutations, suggesting that this adaptation may not be related to the antibiotic class mechanism of action.

We next examined whether mutations in multidrug-resistant strains also converged to specific adaptation mechanisms. To perform this analysis, we tested for repeatedly mutated genes that were exclusively found in evolved strains determined to be broad antibiotic resistant by our drug sensitivity assays (Fig. 2B). The results of this analysis are presented in Fig. 4B. In agreement with the mutation frequency analysis (Fig. 3B), we observed that adaptive mutations in multidrug-resistant strains converged to a key efflux pump regulator encoded by the *acrR* gene. However, this analysis highlighted adaptations in two additional regulatory genes, *lon* and *marR,* of the AcrAB-TolC efflux pump. This efflux pump belongs to the resistance-nodulation-division (RND) family that is known to recognize a broad range of substrates, including antibiotics (33).

The impact of these adaptive mutations on the regulation of the AcrAB-TolC efflux pump is summarized in Fig. 4C. The *acrR* gene is the transcriptional repressor of the *acrAB* operon (34). In our multidrug-resistant strains, adaptive mutations in *acrR* were present in four of the five nonantibiotic conditions and in two of the three antibiotic conditions. In total, *acrR* was mutated in 19 out of 32 multidrug-resistant strains (Fig. 4C). The mutations in *acrR* were primarily in the coding region (92.9%). The effect of these mutations in the coding region was roughly evenly split between loss-of-function and functional modifications. A reduction or loss of *acrR* activity is expected to result in upregulation of the AcrAB-TolC efflux pump, increasing drug efflux and resistance.

The *lon* gene encodes a protease that degrades MarA, an inducer of the AcrAB-TolC efflux pump (35). Adaptive mutations in *lon* were identified in one nonantibiotic condition and one antibiotic condition. In total, *lon* was mutated in 6 out of 32 multidrug-resistant strains (Fig. 4C). All cases of *lon* mutations were IS*186* insertions into a known hotspot in the promoter region (36, 37). This insertion is expected to reduce Lon-mediated degradation of MarA, leading to upregulation of the AcrAB-TolC efflux pump. The *marR* gene is another key regulator of the efflux pump. In our multidrug-resistant strains, adaptive mutations in *marR* were only selected after exposure to the antibiotic chloramphenicol.

Taken together, multidrug resistance arising from selection by both antibiotics and nonantibiotics repeatedly converged on a common resistance mechanism, upregulation of a multidrug efflux pump. This upregulation is primarily driven by mutations in *acrR,* which is expected to reduce the repression of efflux pump expression, while a smaller subset of mutations in the *lon* promoter and in the *marR* gene likely also acted by increasing efflux pump expression. The mutations in these regulators were not exclusive and were observed together in evolved strains. For example, two sertraline evolved strains had mutations in both *acrR* and *lon*.

## DISCUSSION

Previous studies have anecdotally shown that exposure to nonantibiotics, such as antidepressants (6, 9–11) and antipsychotics (8), can select for multidrug resistance through mechanisms such as efflux pump upregulation. These findings demonstrate that drugs not intended to target bacteria can nonetheless still drive resistance phenotypes that extend across antibiotic classes. However, a systematic understanding of how frequently such cross-resistance arises, especially in comparison to antibiotics, remains lacking. Here, we systematically surveyed resistance evolution across a panel of forty drugs in a single and well-controlled model system. Our drug panel consisted of 21 nonantibiotics with different clinical applications (Fig. 1B), and this panel was selected from 1,054 nonantibiotics previously tested for antibacterial activity (6). We compared these nonantibiotics to a panel of 19 antibiotics representative of the eight major antibiotic classes (Fig. 1B). By directly comparing antibiotics and nonantibiotics under identical experimental conditions, our approach enables quantification of how frequently multidrug resistance arises from both antibiotic and nonantibiotic exposures.

Our results revealed two key insights into the evolution of multidrug resistance under both antibiotic and nonantibiotic selection. First, we found that the emergence of multidrug resistance was relatively infrequent and occurred at comparable rates across both drug groups (Fig. 2B). This observation suggests that despite differences in clinical use and primary targets, most drugs do not readily select for multidrug resistance as an initial adaptation. Comparison to previous studies on antibiotic resistance in lab evolution experiments (12–14) revealed that the antibiotics we highlighted—chloramphenicol, tetracycline, and ciprofloxacin—were previously observed to lead to multidrug resistance. However, previous publications also noted multiple additional antibiotics leading to multidrug resistance as well as multiple cases of collateral sensitivity. These wider trends likely arose from differences in the experimental protocols, including longer evolution periods or stronger selection pressure, that allowed for the accumulation of multiple independent adaptations.

The second insight revealed from our work is related to the convergence of evolutionary paths toward multidrug resistance observed in more than 25 independently evolved strains. Whole-genome sequencing revealed repeated mutations in two regulatory genes, *acrR* and *lon*, across independently evolved populations (Fig. 4B and C). This convergence suggests that evolution toward multidrug resistance resulted from a limited number of adaptive solutions. Mechanistically, mutations in these genes are known to result in upregulation of the AcrAB-TolC multidrug efflux pump, thereby increasing the efflux of diverse compounds and conferring broad antibiotic cross-resistance (33, 34, 36–38). Upregulation of efflux pumps may also serve as a stepping stone for acquiring more adaptations by increasing the mutation rate. Specifically, overexpression of efflux pumps suppresses the expression of the mismatch repair protein MutS and thereby increases the rate of spontaneous mutations (39). We note that the mutation pattern we observed is different from adaptation patterns previously reported for antipsychotic- and antidepressant-induced resistance, which identified mutations in *marR* as one of the major drivers of efflux-mediated multidrug resistance (6, 8–11). Taken together, our findings suggest that while multidrug resistance is rare, its emergence is governed by predictable and conserved regulatory mechanisms.

Overall, our work provides a systematic framework for evaluating the risk of multidrug resistance arising from nonantibiotic exposure. While the overall frequency of cross-resistance was low, our results suggest that this risk still exists and should therefore be further evaluated in a more physiologically relevant context. First, our work focused on a lab strain of *E. coli* and not a clinical isolate. Second, laboratory evolution in a single-species system does not capture the complexity of microbial communities, host interactions, and pharmacokinetics that shape drug exposure *in vivo*. The results from our work can be used to prioritize which nonantibiotics should be used for further *in vivo* studies. Future studies should therefore extend this work to more natural settings, including *in vivo* models of the gut microbiome, where chronic exposure to nonantibiotics may impose sustained selective pressures. Mouse models could be used to directly test whether prolonged treatment with commonly prescribed nonantibiotics, such as the antidepressant sertraline, promotes the emergence of multidrug resistance in complex microbial communities. In parallel, retrospective analyses in human cohorts offer a complementary approach to assess whether the use of nonantibiotics increases the prevalence of antibiotic-resistant bacteria. Recent work has found an association between exposure to proton-pump inhibitors, beta-blockers, and antimetabolites and the increased prevalence of antibiotic-resistant infections in patients (40). Together, such approaches will be essential for determining the clinical relevance of nonantibiotic-driven resistance and for guiding strategies to mitigate unintended contributions to antibiotic resistance.

## MATERIALS AND METHODS

### Bacterial strains, media, and growth conditions

The bacterial strain used in this study was *E. coli* MG1655 ΔlacZ::str^r^-barcode ΔgatZ. All growth experiments were performed in defined minimal synthetic M9 media: M9 salts (Fisher Cat#DF0485-17), supplemented with 0.2% amicase (Sigma Cat#82514-1KG) and 0.4% glucose (Fisher Cat#D16-1). Overnight cultures for all growth experiments were grown at 37°C with orbital shaking at 200 rpm with 30 μg/mL of streptomycin antibiotic.

### Lab evolution experiment

We evolved drug-resistant bacteria using a serial transfer protocol. We started the experiment by inoculating each barcoded strain from frozen stock and grew them overnight in M9 media with 30 μg/mL of streptomycin. All replicates shared the same background of *E. coli* MG1655 ΔgatZ ΔlacZ::str^r^, with each having a unique barcode for a total of 168 barcoded strains. We normalized the cultures to an OD600 of 1 and diluted them 1:200 into wells of 96-deep well plates containing the 1 mL M9 media supplemented with the drug at the concentration that inhibits growth by 50% (IC50). The drug panel for evolution is in Table 1. The drug was first diluted from the stock solutions into DMSO or water before being added to M9 media to maintain consistent solvent concentrations across conditions. We inoculated four wells for each drug and four wells for each solvent control (DMSO and water) for a total of 168 wells. We grew the cultures at 37°C and orbital shaking at 200 rpm and diluted the cultures 1:200 into fresh media daily, either with or without drug. We increased the drug concentration by 1.5-fold every fourth passage. After completing 23 transfers, we froze samples as 20% glycerol stocks at −80°C. We isolated individual clones from each frozen stock by streaking evolved populations on LB agar plates and inoculating a single colony for further growth in a liquid culture with M9 media.

**TABLE 1:** Drug panel for evolution

Type	Drug	Clinical class	Catalog no.	Manufacturer	CAS no.	Solvent
Nonatibiotic	2-Thiouracil	Antithyroid	HY-B0503	MedChem Express	141-90-2	DMSO
Nonatibiotic	Azacitidine	Antimetabolite anticancer	HY-10586	MedChem Express	320-67-2	DMSO
Nonatibiotic	Bleomycin	DNA-damaging anticancer	BP971	MilliporeSigma	9041-93-4	Water
Nonatibiotic	Carmofur	Antimetabolite anticancer	HY-B0182	MedChem Express	61422-45-5	DMSO
Nonatibiotic	Chloroxine	Antifungal	HY-B0295	MedChem Express	773-76-2	DMSO
Nonatibiotic	Ciclopirox	Antifungal	HY-B0450A	MedChem Express	9041-93-4	DMSO
Nonatibiotic	Cisplatin	DNA-damaging anticancer	HY-17394	MedChem Express	15663-27-1	DMSO
Nonatibiotic	Deferoxamine	Iron chelator	HY-B0988	MedChem Express	138-14-7	Water
Nonatibiotic	Dichlorophene	Anthelmintic	HY-12638	MedChem Express	97-23-4	DMSO
Nonatibiotic	Diiodohydroxyquinoline	Antiprotozoal	HY-B1400	MedChem Express	83-73-8	DMSO
Nonatibiotic	Doxifluridine	Antimetabolite anticancer	HY-B0021	MedChem Express	3094-09-5	DMSO
Nonatibiotic	Floxuridine	Antimetabolite anticancer	HY-B0097	MedChem Express	50-91-9	DMSO
Nonatibiotic	Flucytosine	Antifungal	HY-B0139	MedChem Express	2022-85-7	DMSO
Nonatibiotic	Mercaptopurine	Antimetabolite anticancer	HY-13677	MedChem Express	50-44-2	DMSO
Nonatibiotic	Mitomycin C	DNA-damaging anticancer	S8146	SelleckChem	50-07-7	DMSO
Nonatibiotic	Pentamidine	Antiprotozoal	HY-B0537B	MedChem Express	140-64-7	DMSO
Nonatibiotic	Sertraline	Antidepressant	HY-B01764	MedChem Express	79559-97-0	DMSO
Nonatibiotic	Streptozotocin	DNA-damaging anticancer	HY-13753	MedChem Express	18883-66-4	DMSO
Nonatibiotic	Thioguanine	Antimetabolite anticancer	HY-13765	MedChem Express	154-42-7	DMSO
Nonatibiotic	Trifluridine	Antiviral	HY-A0061	MedChem Express	70-00-8	DMSO
Nonatibiotic	Zidovudine	Antiviral	HY-17413	MedChem Express	30516-87-1	DMSO
Antibiotic	Azithromycin	50S	A2076	TCI America	83905-01-5	Water
Antibiotic	Aztreonam	Cell wall	A2466	TCI America	78110-38-0	DMSO
Antibiotic	Carbenicillin	Cell wall	BP2648	Fisher Scientific	4800-94-6	Water
Antibiotic	Cefdinir	Cell wall	C3111	TCI America	91832-40-5	DMSO
Antibiotic	Chloramphenicol	50S	227920250	Acros Organics	56-75-7	Water
Antibiotic	Chlorhexidine	Cell membrane	461020010	Thermo Scientific	55-56-1	DMSO
Antibiotic	Ciprofloxacin	DNA	J61317	Thermo Scientific	85721-33-1	Water
Antibiotic	Clarithromycin	50S	C22201G	TCI America	81103-11-9	DMSO
Antibiotic	Colistin	Cell membrane	455392500	Thermo Scientific	1264-72-8	Water
Antibiotic	Enoxacin	DNA	J61912	Thermo Scientific	74011-58-8	Water
Antibiotic	Furazolidone	Nitrofuran	F9505	Sigma-Aldrich	67-45-8	DMSO
Antibiotic	Kanamycin	30S	J60668	Alfa Aesar	25389-94-0	Water
Antibiotic	Nitrofurantoin	30S	N0883	TCI America	67-20-9	DMSO
Antibiotic	Norfloxacin	DNA	N0817	TCI America	70458-96-7	Water
Antibiotic	Rifampicin	RNA	HY-B0727/ CS-2261	MedChem Express	13292-46-1	DMSO
Antibiotic	Sulfamethoxazole	Folic acid	S7507	Sigma-Aldrich	723-46-6	DMSO
Antibiotic	Sulfamonomethoxine	Folic acid	S0592	TCI America	1220-83-3	DMSO
Antibiotic	Tetracycline	30S	B21408	Thermo Scientific	64-75-5	Water
Antibiotic	Trimethoprim	folic acid	T7883	Sigma-Aldrich	738-70-5	DMSO

### Monitoring growth curves

We grew drug-evolved clones overnight in M9 media with 30 μg/mL of streptomycin. We diluted bacteria to an OD600 of 0.005 and transferred them to 96-well plates (Nunc Cat# 167008) containing serial dilutions of their respective drug in triplicate to reach a final volume of 120 μL. The concentration range and the dilution series were chosen to capture multiple concentrations with sub-inhibitory effects (typically 3–5 concentrations). For some drugs, we used adjusted ranges and dilution series for evolved strains to capture increased resistance (Table S1). This approach enabled consistent comparison of resistance levels across drugs and replicates. We incubated the cultures at 37°C with 800 rpm shaking and determined the absorbance (600 nm) after 8 h of growth using an automated microplate reader (BioTek LogPhase 600). Using a custom MATLAB (MathWorks) script, we first divided the absorbance by the absorbance value of the no-drug control to quantify growth inhibition. Then, we fit a logistic curve to the mean absorbance ratio of the biological replicates, generated dose-response curves, and inferred the concentration that inhibits growth by 50% (absolute IC50). For strains that did not exhibit 50% growth inhibition within the tested range, the IC50 was set to the maximum tested drug concentration. Drug concentration ranges are in Table S1. We compared the sensitivity of the ancestor to the evolved replicates using a one-tailed Welch’s *t*-test to check whether the evolved replicates had consistently higher IC50 than the ancestor (alpha = 0.05). We used this same procedure to test the ancestor strain across all drugs to determine the baseline IC50 for the first day of the evolution experiments.

### Cross-resistance of evolved clones

We evaluated the antibiotic cross-resistance of the clones, in comparison to the ancestor, with a panel of 21 antibiotics that represent eight major antibiotic classes. We followed the recommended EUCAST (20) guidelines for broth microdilution assay (BMD) with some modifications. We prepared drug plates in 96-well U-bottomed plates (Fisher, Cat #FB012932) with 25 μL of M9 media supplemented with a single antibiotic. The plates contained serial dilutions at 1:2 or 1:4 of the tested drugs in four concentrations along with four wells of no drug control. The dilution ranges were chosen based on preliminary experiments (Table 2). The drug plates were stored at −20°C until use. We grew the evolved clone and the ancestor overnight in M9 media with 30 μg/mL of streptomycin and normalized the culture to 1 × 10^6^ CFU/mL. We then diluted each culture 1:2 to a final concentration of 5 × 10^5^ CFU/mL, into three replicate drug plates. We placed the broth microdilution plate in a 37°C standing incubator for 24 h and then scanned the plate using a flatbed document scanner (Epson Perfection V750 Pro scanner in photo mode, 8-bit grayscale, and 150 dpi resolution).

**TABLE 2:** Drug concentrations for broth microdilution

Drug	Class	Catalog no.	Manufacturer	CAS no.	Lowest concn (μM)	Lowest concn (μg/mL)	Highest concn (μM)	Highest concn (μg/mL)	Dilution factor
Kanamycin	30s	J60668	Alfa Aesar	25389-94-0	7.81	4.55	500	291.30	4
Nitrofurantoin	30s	N0883	TCI America	67-20-9	15.63	3.72	1,000.00	238.16	4
Tetracycline	30s	B21408	Thermo Scientific	64-75-5	0.98	0.47	7.81	3.76	2
Azithromycin	50s	A2076	TCI America	83905-01-5	3.91	2.93	250	187.25	4
Chloramphenicol	50s	227920250	Acros Organics	56-75-7	7.81	2.52	500	161.57	4
Clarithromycin	50s	C22201G	TCI America	81103-11-9	62.5	46.75	500	373.98	2
Chlorhexidine	Cell membrane	461020010	Thermo Scientific	55-56-1	1.95	0.99	15.63	7.90	2
Colistin	Cell membrane	455392500	Thermo Scientific	1264-72-8	1.56	2.18	12.5	17.51	2
Aztreonam	Cell wall	A2466	TCI America	78110-38-0	0.02	0.01	1.56	0.68	4
Carbenicillin	Cell wall	BP2648	Fisher Scientific	4800-94-6	3.91	1.48	250	94.60	4
Cefdinir	Cell wall	C3111	TCI America	91832-40-5	0.39	0.15	25	9.89	4
Ciprofloxacin	DNA	J61317	Thermo Scientific	85721-33-1	0.02	0.01	1.56	0.52	4
Enoxacin	DNA	J61912	Thermo Scientific	74011-58-8	0.2	0.06	12.5	4.00	4
Norfloxacin	DNA	N0817	TCI America	70458-96-7	0.1	0.03	6.25	2.00	4
Sulfamethoxazole	Folic acid	S7507	Sigma-Aldrich	723-46-6	7.81	1.98	62.5	15.83	2
Sulfamonomethoxine	Folic acid	S0592	TCI America	1220-83-3	3.91	1.10	250	70.08	4
Trimethoprim	Folic acid	T7883	Sigma-Aldrich	738-70-5	1.95	0.57	125	36.29	4
Furazolidone	Nitrofuran	F9505	Sigma-Aldrich	67-45-8	1.95	0.44	125	28.15	4
Rifampicin	RNA	HY-B0727/CS-2261	MedChem Express	13292-46-1	4.88	4.02	39.06	32.14	2
Rifabutin	RNA	AC461210010	Thermo Scientific	72559-06-09	7.81	6.62	62.5	52.94	2
Cetrimonium bromide	Cell membrane	227160100	Thermo Scientific	57-09-0	15.63	5.70	125	45.56	2

We analyzed the resulting BMD plate images using a computational approach we previously developed (21). We used a convolutional neural network pre-trained on BMD plate images of *E. coli* growth to evaluate the pellet sizes in the BMD plate images. Our script classified images into distinct image classes that indicated robust, marginal, or no growth. We reviewed every automated phenotype call made by the script and made manual corrections for misclassified growth phenotypes. We defined the MGC as the highest concentration at which robust growth was observed. We used this metric since it can better capture small changes in drug resistance (in contrast to standard MIC measurements that span concentration ranges by 2-fold increases). If a well with marginal growth is followed by a well with robust growth at the next lowest concentration, we averaged the concentrations in these two wells to calculate the MGC. We considered anything within 2-fold of the ancestor to be within the error of the assay and adjusted it to indicate no change. We considered a strain to be resistant to the antibiotic for any MGC increase that was 2-fold or greater. An evolved strain was considered sensitive to an antibiotic if its MGC was 2-fold lower or below that.

To infer the radar plots that summarize antibiotic cross-resistance in evolved strains, we took the following steps. For each evolved strain, we inferred a binary vector of resistance across all 21 representative antibiotics (using the 2-fold resistance cutoff explained above). We then calculated for each antibiotic class the proportion of evolved replicates that were resistant to antibiotics from that class only. A proportion of one indicates that all evolved replicates become resistant to all antibiotics from that specific antibiotic class. We used a similar approach to infer radar plots that summarize antibiotic cross-sensitivity.

### Whole genome sequencing

We extracted genomic DNA from each evolved strain using the Zymo Quick DNA Fungal/Bacterial Mini-prep kit (Zymo Research, Cat# D6005). DNA sequencing was performed by SeqCoast Genomics, LLC (Portsmouth, NH, USA). SeqCoast prepared the library using the Illumina DNA Prep kit and IDT 10 base-pair UDI indices. Sequencing was performed on an Illumina NextSeq 2000 instrument (2 × 150 bp). Read demultiplexing, read trimming, and run analytics were performed using DRAGEN v3.10.12, the on-board analysis software of the NextSeq2000. We used the breseq tool (41) to align the reads to the reference genome (NCBI accession: NC_000913) and identify the mutations and genomic rearrangements. Using a custom Python script, we determined the coverage for each position. Using the coverage and a custom MATLAB (MathWorks) script, we determined the gene amplifications for each genome. We considered junction and missing coverage evidence as predicted mutations because breseq cannot resolve mutations involving IS elements and insertion sequences to the nucleotide level; hence, it categorizes them as evidence instead of a predicted mutation. We used a custom MATLAB script to remove the mutations in a representative ancestral strain from the drug-evolved clones. We also removed mutations present in all the strains, including the controls. Mutations that were removed are detailed in Table S3 (sheet 2: Mutations Removed). The script compiled the mutations across the four evolved clones per drug and mapped the mutations to genomic coordinates for visualization.

## Data Availability

All computer code and the data sets associated with this study were uploaded to GitHub (https://github.com/Mitchell-SysBio/2026_NonAntibiotic) and Zenodo (https://doi.org/10.5281/zenodo.21283927). The whole genomes of evolved bacteria are available on the NCBI Sequence Read Archive under Bioprojects PRJNA1463147.

## SUPPLEMENTAL MATERIAL

Supplementary Figures mbio.01168-26-s0001.pdf Figures S1–S3.

Table S1 mbio.01168-26-s0002.xlsx Auto-resistance and cross-resistance.

Table S2 mbio.01168-26-s0003.xlsx Mutations determined by whole genome sequencing.

Table S3 mbio.01168-26-s0004.xlsx Predicted mutations determined by breseq software.

Table S4 mbio.01168-26-s0005.xlsx Junction evidence determined by breseq software.

Table S5 mbio.01168-26-s0006.xlsx Missing coverage evidence determined by breseq software.

Table S6 mbio.01168-26-s0007.xlsx Amplification evidence determined using breseq analysis pipeline.

ASM does not own the copyrights to Supplemental Material that may be linked to, or accessed through, an article. The authors have granted ASM a non-exclusive, world-wide license to publish the Supplemental Material files. Please contact the corresponding author directly for reuse.
