# Supplementary material for: Nonantibiotic-driven evolution reveals rare but predictable routes to broad antibiotic resistance
Source: mBio. 2026 Aug 3;17(9):e01168-26. doi: 10.1128/mbio.01168-26 (PMC13556239; doi:10.1128/mbio.01168-26)
Supplement: Supplementary file 1 [file mbio.01168-26-s0001.pdf]

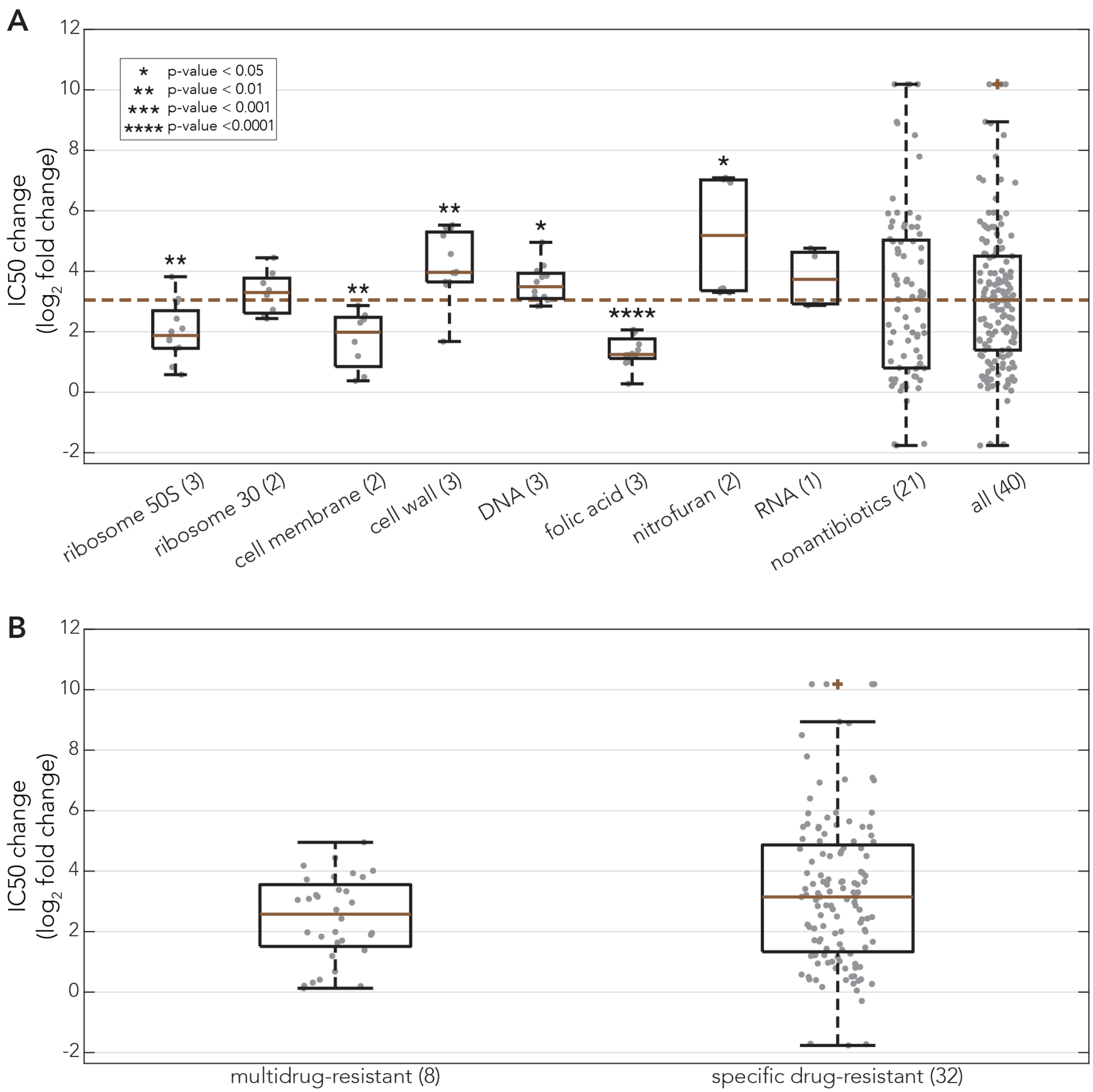


**Supplementary Figure 1. Auto-resistance by drug type**. **A.** The change in auto-resistance in evolved strains categorized by different drug groups in comparison to all strains. Auto-resistance of individual strains are shown as grey circles. The star indicates statistical significance in a t-test comparing the mean of the group to the median of all strains. The number in the label is the number of drugs in that group. **B.** Multidrug resistance is not associated with higher levels of auto-resistance. Strains are grouped by their cross-resistance profiles into multidrug-resistant strains and specific drug-resistant strains. A t-test comparing the group means was not statistically significant (p-value = 0.0562). Auto-resistance of individual strains are shown as grey circles.

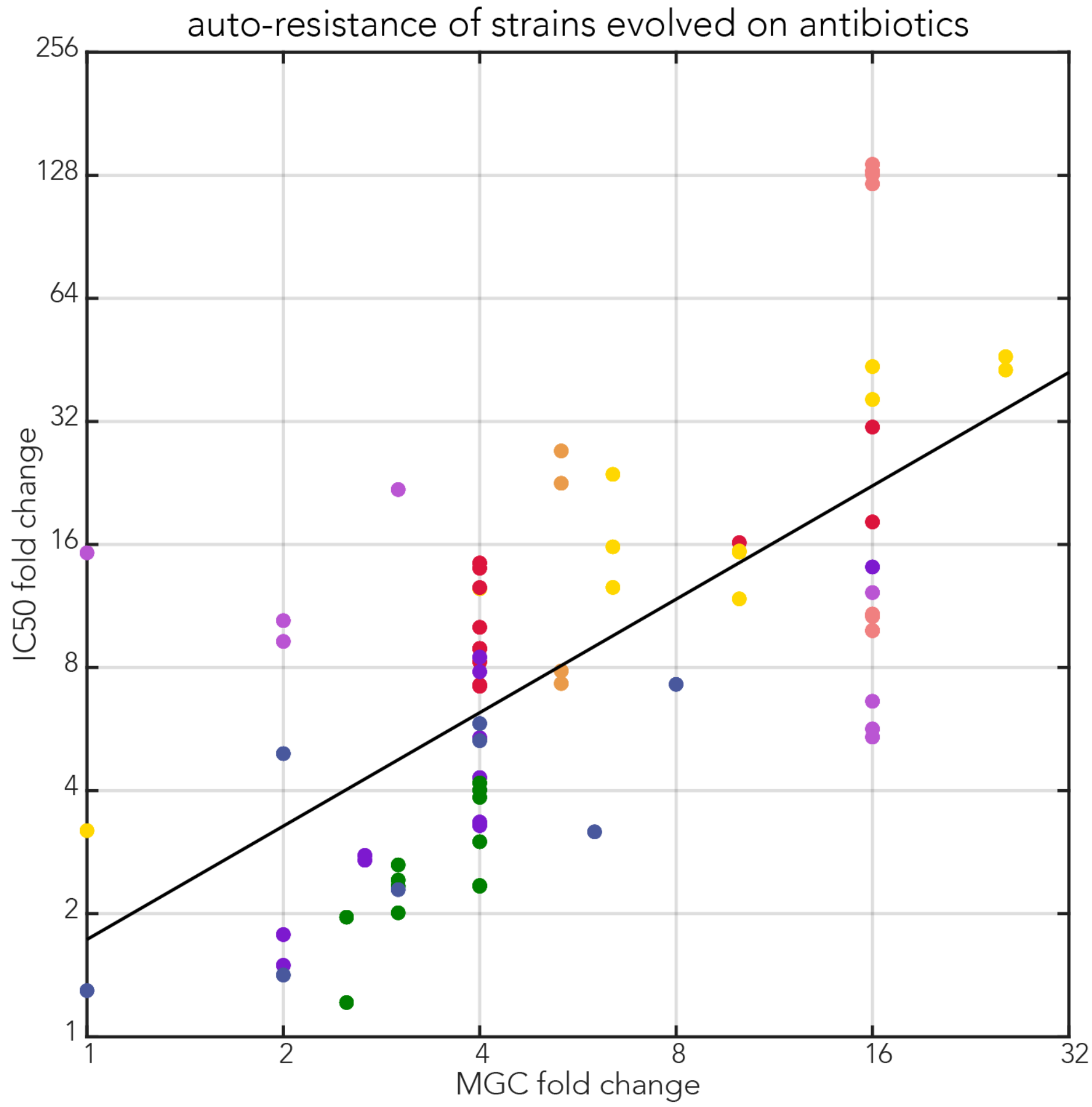


**Supplementary Figure 2. Auto-resistance measured by the dose-response assay and the broth microdilution assay are correlated**. The dots mark the resistant levels of 76 individual antibiotic evolved strains measured separately by the two assays. Each dot marks the mean of three technical replicates. The colors correspond to the antibiotic class. The black line marks the Pearson correlation fit ($r = 0.67$, $p = 5.25 \times 10^{-11}$).

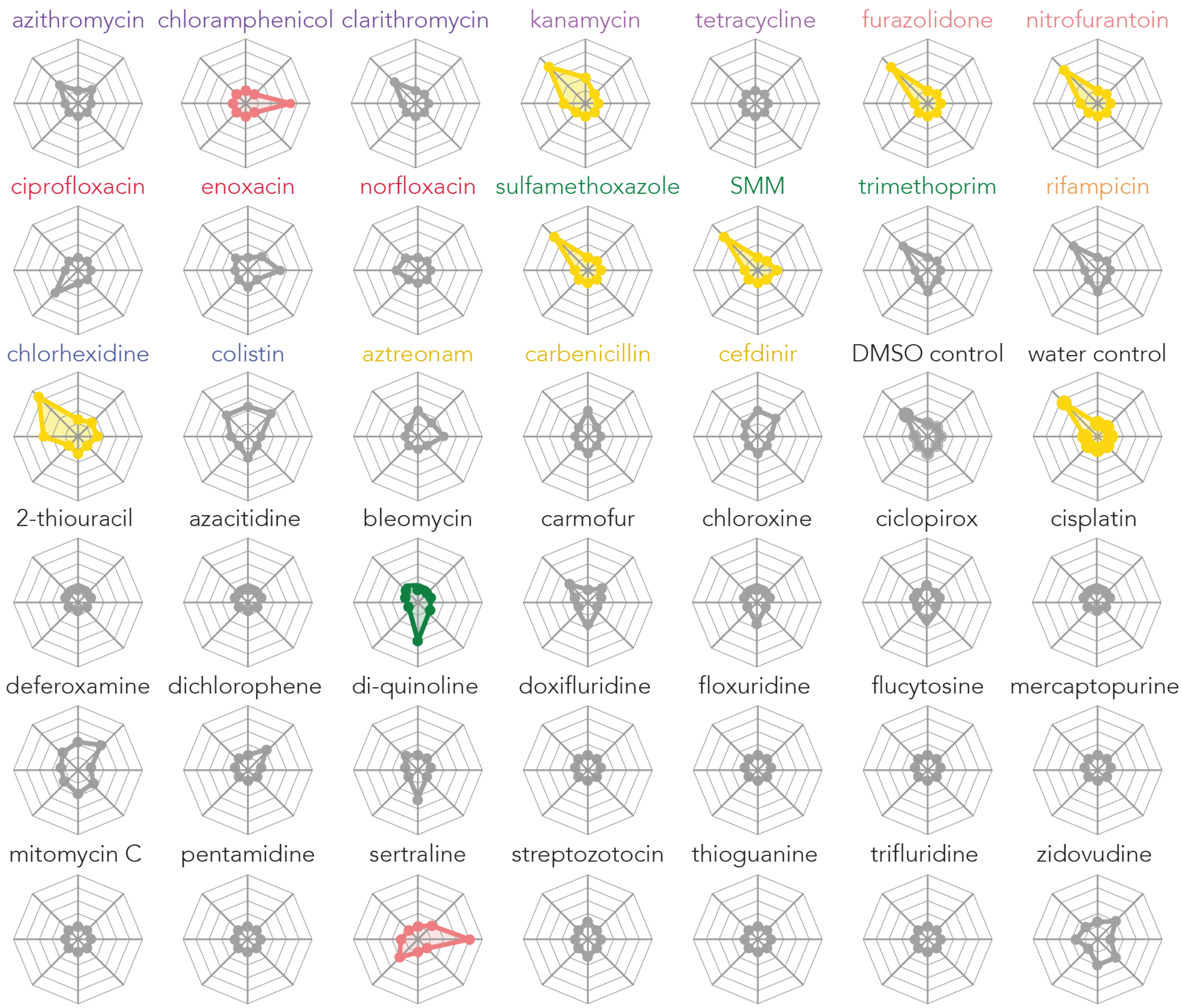


**Supplementary Figure 3. Sensitivity profile across the drug panel.** The sensitivity profile of strains evolved on the forty drugs. The color of the web denotes the antibiotic class the strain is most sensitive to, color of the drug name marks the class of the drug used for evolution. An empty web indicates no sensitive classes. Sulfamonomethoxine is abbreviated as SMM and diiodohydroxyquinoline is abbreviated as di-quinoline.
